# Supplementary material for: Are carbon emissions trading and green financial instruments synergistic? -Comprehensive quantitative research based on content analysis
Source: PLoS One. 2024 Mar 7;19(3):e0298601. doi: 10.1371/journal.pone.0298601 (PMC10919601; doi:10.1371/journal.pone.0298601)
Supplement: S1 Data — (ZIP) [file pone.0298601.s001.zip › Data/地级市Z_F工作报告.zip!/╣π╢1⁄2/├»├√╩╨/└┤╫╘╣1⁄2╓┌║┼í╛┬φ┐╦╩2╛▌═°í┐_2016.docx]

各位代表：

　　我代表市人民政府向大会作工作报告，请予审议，并请政协委员和其他列席人员提出意见。

一、“十二五”时期工作回顾

过去五年，是我市发展征程中不平凡的五年。面对经济下行压力增大以及系列重大事件冲击等困难挑战，在省委、省政府和市委坚强领导下，在市人大及其常委会和市政协监督支持下，市政府坚决贯彻落实全省进一步促进粤东西北地区振兴发展决策部署，始终把加快发展作为解决所有问题的关键，迎难而上、主动作为，不仅有效应对了各种风险挑战，而且较大地提升和发展了茂名，完成了“十二五”规划的主要目标任务。

（一）坚持发展第一要务，经济实力跃上新台阶。

　　“十二五”时期，我市聚精会神搞建设，一心一意谋发展，激发发展活力，破解发展瓶颈，拉开发展格局，形成发展热潮，办成了系列实事难事，极大增强了全市的信心。

　　综合竞争力提升。2015年，全市实现地区生产总值2445.6亿元，增长8.0%，五年年均增速10.6%，经济总量稳居粤东西北地区首位；公共财政预算收入113.9亿元，增长10.6%，五年间翻了一番多；固定资产投资完成1115.5亿元，五年年均增速50.1%，总量从全省倒数跃升至中游。金融服务发展能力明显提升，存贷比五年提升8.3个百分点。在省对粤东西北地区振兴发展评估考核中，2013、2014年连续两年获得总分第一。

　　工业实力显著增强。2015年，全市实现规上工业增加值713亿元，五年间翻了近一番。新增134家规上工业企业。新增高州、化州2个省级产业转移工业园，在全省率先实现省级工业园县域全覆盖。茂名石化炼油改扩建工程完工，原油加工能力达2000万吨/年。茂名热电厂“上大压小”扩建工程竣工投产。成功引入德国巴斯夫异壬醇、中海油粤西LNG和联塑建材等大型企业项目。

　　现代农业发展成果丰硕。五年新增省级以上农业龙头企业37家，建成国家级农民专业合作示范社30家。建成怀乡鸡国家级农业标准化示范区和水东芥菜省级标准化示范区。投入资金20多亿建设现代高标准农田114.6万亩。粤西农批水果和蔬菜交易区正式运营。全国500强海亮集团千亩种植基地、全球首个桂圆电子交易中心落户高州。

发展新动力加快培育。电子商务发展迅猛，建成淘宝和京东县级服务中心6个、村级服务站191个。2015年全市快递业务量同比增长64.9%。商贸物流业发展较快，亿丰家居、华南商贸城和市交投物流园等一批重大项目进展顺利，市民高新片区商贸物流集聚区基本成形。六韬珠宝等兼具工业地产和孵化器功能的项目加快推进。

（二）坚持交通先行，基础保障能力全面加强。

　　交通建设进入集中开工、大步发展时期，立体化交通网络初步构建，五年累计投资267亿元，是“十一五”时期的2.7倍。环保、水利、电力、通信等基础设施建设全面推进。

　　对外交通跨越式发展。包茂高速茂名段建成通车，全市高速公路通车里程达200公里。汕湛高速茂名段全面开工。云茂高速前期工作加快推进。茂湛铁路建成通车。深茂铁路江门至茂名段开工建设。湛江机场迁建已确定首选场址。茂名综合客运枢纽和博贺疏港铁路前期工作加快推进。

　　对内交通加速一体化。改造完成茂名大道、茂高快线、茂化快线，“两区两市”纳入中心城区半小时生活圈。茂名港大道、海洋大道东段、悦民路南段、环城东路、西城西路、高长线二期等新建项目建成通车。茂东快线、市民大道二期基本建成。完成国省道改造工程18项596.3公里，G207、G325以及S280、S282、S284、S370等15条国省道相继完成改造。完成新农村公路路面硬化3290公里。

　　环境治理能力有效提升。“城乡清洁工程”五年投入超过10亿元，市生活垃圾焚烧发电厂、各区（市）垃圾填埋场均建成投用；110个镇（街）建成生活垃圾转运（压缩）站。河西城区生活污水处理厂以及信宜、电白、高州金山工业园区污水处理厂建成投用。茂南污水处理厂即将建成。

水利电力工程扎实推进。五年水利建设投资57.6亿元，增长92%。高州水库除险加固工程基本完成；高州水库灌区续建配套节水改造首期工程建成投用。建成名湖水库至河东水厂第二输水管线。滨海新区供水工程和水东湾城区引罗供水工程开工建设。电网结构大幅优化提升，实现主网220千伏双环网格局。

（三）坚持滨海发展战略，构建港业城联动发展新格局。

　　以滨海新区“三大平台”为主战场，推动港业城联动发展，初步形成滨海发展新格局。

　　滨海新区起步区建设全面推进。深水大港雄姿初现，东防波堤推进至3300多米，西防波堤推进至1100多米。粤电煤炭码头工程基本建成。广州港集团博贺通用码头正抓紧开工前准备工作。粤电燃煤电厂项目基本建成。博贺湾片区规划建设同步推进，博贺湾大道、博贺湾大桥等基础设施建设加快推进。

　　高新区产业基础更加坚实。五年建成项目68个，投资76.7亿元；在建项目56个，投资81亿元。高新区北片区开发成型，西南片区全面启动建设。产业多元化发展，健康产业园区、光伏发电项目等有序推进。国家危险化学品质量监督检验中心（广东）落户高新区。获批国家新型城镇化试点。全面完成乙烯厂卫生防护距离搬迁安置工作，彻底解决该厂“久试未验”问题。

　　水东湾新城建设初具规模。水东湾综合整治开发项目进展顺利，跨海大桥进入全面施工阶段，水东湾清淤完成一期工程，海洋公园（一期）和滨湾路绿道项目开工。南海旅游岛道路及两侧改造项目启动。高地智慧城路网建设加快推进，歌美海公园和市中心医院项目开工建设。

　　（四）坚持扩容提质，城镇化水平有新提升。

　　坚持向东向南靠海发展战略，中心城区发展框架拉开，人居环境改善，集聚能力增强。

　　中心城区承载能力大幅提升。原茂港区、电白县合并设立电白区。北组团东北五小区、油城十路片区、站南片区和西城片区全面开发。中组团市民片区加快建设。南组团水东湾城区旦场、高地、南海等片区不断拓展。

　　城市环境质量稳步提升。全面推行城市精细化管理，市容环境明显好转。建成南香公园和榕园。打造茂名特色的露天矿生态公园。启动建设官渡公园。基本完成市区公园（广场）湖水治理工程。加强市区大气治理，空气质量指数排名居全省前列。电白城区基本完成东、西湖改造工程和海堤绿道系统改造一期工程。河西老工业区纳入全国老工业区搬迁改造试点，炼油厂卫生防护距离搬迁安置完成一期第一批搬迁工作。

　　中心城区交通加快改善。建成双山七路、西粤南路、官山五路和官渡三路。基本建成官渡四五六路和西粤北路，开工建设文明北路和大园三路。完成高山桥改造工程。完成光华南路、人民路、油城路等20多条道路沥青罩面工程。站南片区改造城南路、金城东路、锦华路二街，新建人民南五六路；西城片区进园路、周坑大道等加快推进。电白城区加快推进“七路一桥一广场”建设，厂前路、人民路完成改造，新湖路改造加快推进。开通北组团至南组团和东组团公交线路。

县域城区提质升级。信宜获评国家生态保护与建设示范区，重点推进站前片区开发建设，打造锦江河“一河两岸”景观带，建成淘金湾广场；高州市重点建设“七路一桥一中心”，建成东方大道、笔架山森林公园等重点工程；化州市重点加快“三江六岸”和“三大出口”项目建设。

（五）坚持改革发展，体制机制焕发新活力。

　　坚持问题导向，突出重点领域和关键环节改革，经济社会发展活力不断增强。

　　深入推进行政体制改革。新一轮行政审批制度改革以来累计调整行政审批事项440项，调整比例达48.6%。市县镇村四级行政服务中心和网上办事大厅全面建成。商事制度改革有序推进。基本完成新一轮政府机构改革。完成事业单位分类改革。推进重点领域综合行政执法体制改革。完成市、县政府工作部门权责清单编制和公布工作。

　　深化经济体制改革。健全财政预算管理体制，市级试行编制零基预算；集中使用大额资金、清理盘活闲置资金、非税收入分配、政府性债务管理纳入制度化轨道。完成公务用车制度改革，整体节支率达7%。深化国有企业改革，成立市国资委，推进国资监管全覆盖。获批省农村金融改革创新综合试验区。

　　创新投融资模式。推广政府与社会资本合作（PPP）融资模式，市级采用BT融资项目32项。面向民间资本公开招标重大项目。资本市场融资实现突破，拓展了发行企业债券、中期票据、信托计划等融资渠道。

　　创新工作体制机制。滨海新区起步区、高新区推行区镇、园镇互动改革。水东湾新城管理体制逐步理顺。创新工作方式方法，建立和推广包茂高速“大包干”机制和“六个一”督导机制。建立完善“两会两考两通报”制度。

　　（六）坚持民生导向，民生福祉持续改善。

　　加大民生投入，五年民生累计支出919.7亿元，年均增长25.3%。省十件民生实事市县配套资金拨付进度连续三年居全省第一。2015年统筹6000万元公用经费用于重点民生项目支出。

　　全民社保扎实推进。城镇职工基本养老保险覆盖率80.2%。企业退休人员人均基本养老金从2010年1152元/月提至2015年2027元/月。城乡居民养老保险基础养老金提至100元；城乡居民医保筹资标准提至430元。实施城乡居民大病保险制度和城镇职工补充医疗保险。城乡低保五保救助和孤儿基本生活保障水平大幅提高。城乡医疗救助工作全面铺开。

　　民生实事全面加强。就业创业工作有效推进，实现城镇新增就业35.6万人，新增转移就业劳动力54.5万人。建成创业孵化基地8个。养老服务业加快发展，全市新增养老床位1.9万张。建成保障性住房1万多套，完成农村低收入住房困难户住房改造5.8万户。建成粤西救灾物资储备基地。解决234.4万人农村居民饮水不安全问题。圆满完成“9·21”复产重建任务和两轮扶贫开发“双到”工作。

　　社会事业成绩斐然。成功申报国家知识产权试点城市，全市专利申请量是“十一五”时期的5.1倍，获省科技进步奖数量居粤东西北各市前列。教育创强基本完成，投入资金56亿元。建成茂名一中、市二职新校区和愉园中学，市五中实现整体搬迁，开工建设愉园小学。广东石油化工学院校区扩展工作顺利推进，茂名职业技术学院新校区一期工程完工并投用，广州科技职业技术学院滨海校区开工建设，广东茂名健康职业学院成功开办，广东茂名幼儿师范专科学校和广东农林科技职业学院加快筹建，形成“一本五专”高等教育发展新格局。文化事业繁荣发展，市图书馆获评国家一级馆；涌现出一批在全国、全省有影响的精品力作。市博物馆改造提升工程完成。“隋谯国夫人冼氏墓”成为全国重点文物保护单位。群众体育活动异彩纷呈。疫病防控、妇幼保健等公共卫生服务能力持续加强；有效开展H7N9、登革热及埃博拉出血热防控工作。

　　平安建设深入推进。坚持安全发展，着力加强危化品、道路交通、非煤矿山、油气管道等重点领域安全管理，全市安全生产形势总体稳定。茂名危险化学品应急救援基地开工建设。集中开展“六大专项”和“3+ 2”等打击整治行动，全力推动社会治安防控体系建设，有效治理治安重点地区和治安突出问题。率先在全省开展“一村（居）一律师”工作。食品药品安全管理进一步加强。此外，人口计生、民族宗教、国防动员、双拥、统计、气象、地震、残疾人、红十字、外事、侨务、地志、三防、应急、打私、打假等工作都取得新的成绩。

　　切实加强政府自身建设。主动接受人大政协监督、法院检察院监督和社会舆论监督。落实政府常务会议学习制度。加强政务公开。分解落实政府工作报告任务。督办督查工作全面加强。监察、审计监督作用进一步发挥。严格落实中央“八项规定”，整治慵懒散奢和奢靡浪费之风；深入开展群众路线教育实践活动，扎实推进“三严三实”专题教育，打造风清气正、务实担当的政务环境。

　　各位代表！回望过去五年，我们牢牢把握发展主动权，取得了比预期要好的发展成果。我们深深体会到有四点经验弥足珍贵：一是必须坚定信心。面对困难挑战，全市上下始终坚定信心、保持定力是我们战胜困难的第一法宝；二是必须求真务实。强化问题导向，接地气、重心下沉、走群众路线，以实实在在的工作成效取信于民；三是必须敢于碰硬。通过攻克工作中的一个个“硬骨头”，鼓舞士气，昂扬全市干事创业的精神。四是必须锲而不舍。坚持发扬钉钉子精神，对重点工作反复敲打、持续督办、经常过问，最大限度抓落实、保进度、促发展。

　　各位代表！“十二五”时期的发展成就来之不易，这是省委、省政府和市委正确领导的结果，是全市人民团结一致、艰苦奋斗的结果。在此，我代表市人民政府向全市人民，向驻茂人民解放军、武警官兵和各民主党派、人民团体、社会各界人士表示崇高的敬意！向所有关心支持茂名发展的港澳同胞、台湾同胞、海外侨胞和国际友人，表示衷心的感谢！

我们也清醒地看到，我市经济社会发展还面临不少问题：交通仍然是制约发展的主要瓶颈；产业结构过于单一，经济发展方式粗放；增收节支压力大；城市建设、基本公共服务等方面历史欠账较多；发展框架拉开以后，要素需求增加与供应难度加大矛盾突出；机关作风建设有待加强。对此，我们将在今后工作中采取有力措施加以解决。

二、“十三五”时期的发展目标和主要任务

“十三五”时期，是我市厚植发展基础、加快振兴发展战略机遇期。抓好这五年的工作，意义重大。前不久结束的市委十届七次全会，确立了未来五年“一体两翼三大抓手”发展思路和“三个做大”发展战略，为茂名描绘了美好蓝图。我们必须牢固树立创新、协调、绿色、开放、共享的发展理念，紧紧扭住加快发展第一要务，确保全面建成小康社会。

　　“十三五”时期我市经济社会发展主要目标：生产总值年均增长8.5%，人均生产总值年均增长7.5%，城乡居民收入增长与经济增长同步。

　　实现“十三五”规划目标，我们要着力抓好六方面工作：一是构建粤西重要交通枢纽。完善“四横两纵”铁路网。建成“三横三纵”高速路网。打造区域性深水大港。同时，完善提升主干公路网，推进城市组团交通一体化，建设现代客货站场。二是培育壮大主导产业。推动主导产业做大做强，形成新的产业增长点。重点抓好石油化工、农副产品加工、矿产资源及建材、新型金属加工及装备制造、特色轻纺、生物医药与健康、电子商务及生产性服务业等七大主导产业。三是建设粤西组团式中心城市。推进“北优、中联、南拓、东进”四大城市组团建设。优化发展现主城区，中联发展市民高新片区，南拓发展水东湾城区，东进建设博贺湾片区。四是壮大特色县域经济。加快转变农业发展方式，发展现代农业。依托产业园区，引导产业向园区集聚，推进县域产业发展。全面推进新农村建设，推动城乡协调发展。五是深化改革开放，突出抓好行政管理体制、经济体制、金融改革创新。参与“一带一路”建设，加强区域合作，推进外经贸转型升级和招商引资工作。六是着力增进民生福祉。大力推进基本公共服务均等化。加快科技、教育、就业、社保、医疗、扶贫开发等各领域事业。加强生态环境保护，加强和创新社会管理，促进社会和谐稳定。

“十三五”的目标任务已经明确，关键在于抓落实。我们要把规划目标落实到具体工作和具体项目上，落实到部门的工作责任上。要加强宏观谋划，争取更多项目纳入上级“十三五”规划笼子。要进一步完善工作督导机制、推进机制和项目管理机制，扎实推进重点项目建设。我们相信，经过全市人民的不懈奋斗，茂名一定能迎来更加美好的明天！

三、2016年工作安排

今年是全面实施“十三五”规划的开局之年，我们面临的发展环境错综复杂，挑战与机遇并存。一方面，国际市场持续低迷，国内经济步入新常态，区域竞争更加激烈并呈现分化，争先进位难度进一步加大；另一方面，财政金融政策逆周期调节力度加大，钢铁、水泥等大宗产品价格处于低位，发达地区产业转移加快，新产业、新业态蓬勃兴起。为此，要结合茂名发展的阶段性特征，保持发展定力，做到趋利避害，注重把握四个关键：一是牢牢把握做大经济总量这个总前提。我市发展尚处在量变阶段，没有较快增长速度、较大经济总量，就很难为产业调整、城市发展、改善民生提供支撑。二是牢牢把握做大主导产业这个动力源。实业兴茂，不改变主导产业单一局面，就不可能提升我市经济抗风险能力。三是牢牢把握做大城市规模这个大课题。在拉开中心城区框架的同时，要改善环境、完善功能、集聚人气，推动组团融合，增强城市经济规模效应。四是牢牢把握民生福祉这个落脚点。要守住民生底线，把重点放在兜底上，保障群众基本生活和基本公共服务。

　　2016年政府工作总体要求是：全面贯彻党的十八大、十八届三中、四中、五中全会和中央经济工作会议精神，深入学习习近平总书记系列重要讲话精神，认真贯彻落实省委十一届五次、六次全会和省进一步促进粤东西北地区振兴发展工作会议精神，按照市委部署要求，围绕全面建成小康社会目标，坚持“一体两翼三大抓手”发展思路，做大经济总量、做大主导产业、做大城市规模，深入推进交通基础设施建设、产业园区建设和中心城区扩容提质，大力发展特色县域经济，凝心聚力，真抓实干，努力实现“十三五”时期经济社会发展良好开局。

　　今年经济社会发展主要预期目标是：生产总值增长8.5%左右，固定资产投资增长16%，社会消费品零售总额增长10.5%，一般公共预算收入增长9%，居民消费价格涨幅控制在3%以内；居民收入增长与经济增长基本同步，进出口总额和节能减排约束性指标完成省下达年度计划。

　　为此，我们要重点抓好八方面工作：

　 （一）突出抓好交通基础设施建设，加快构筑粤西重要交通枢纽。

　　全力搭建交通“主骨架”。加快汕湛高速茂名段建设。开工建设云茂高速茂名段。加快玉茂一级公路前期工作。完成深茂铁路茂名段征拆收尾工作。加快推进博贺疏港铁路前期工作。加快河茂铁路电气化改造工程。完成博贺新港区东西防波堤主体堤身建设。争取年内运营粤电煤炭码头。开工建设广州港通用码头。推进博贺新港区30万吨航道、30万吨原油码头、保利利丰矿石码头、油品化工码头等前期工作。启动吉达港区建设前期工作。优化提升水东港区功能。加快口岸基础设施建设。

　　加快编织城市组团间交通网。加快推进西部快线、工业大道南段、悦民大道等城市快线建设。优先发展城市公共交通，提高城市公共交通分担率，加强南北组团公交联系。

　　打通交通“毛细血管”。加快完成茂东快线、环市西路路面改造、观珠高速出口至沙琅一级公路（一期）工程。加快县道升级改造。扎实推进危桥改造。完善农村公路网络，完成350公里新农村公路路面硬化任务。做好“十三五”公路建设项目规划和国道路网升级工作。

　　加快建设交通关键节点。开工建设茂名综合客运枢纽。完成火车站改名工作。加快推进（电白）现代铁路货场、湛江机场迁建和粤西空港物流园等项目前期工作。

　　加强道路交通管理。深入落实城乡道路两侧建设实施规划控制的有关规定，整治道路两侧及重点地区违章建筑。坚持严管重罚，加大对超限超载及路面污染执法力度。强化源头治理，严厉查处非法加工、开采矿产砂石行为，严查车辆非法改装窝点，坚决杜绝非法改装车辆上牌、领证和通过年检。

　　（二）突出抓好产业园区建设，加快培育主导产业。

　　抓好工业园区建设。着力推进茂名高新区创建国家级高新区。加快双回路电源供电线路、集中供热、污水处理厂、应急救援基地、循环经济示范中心等项目建设。继续抓好西南片区基础设施和项目建设。积极谋划建设健康产业园、中德精细化工园和现代商贸物流园。抓紧完善博贺新港区物流园区、博贺临港工业区、高地智慧城信息产业园等园区基础设施。进一步完善县域园区体制机制，提升园区用地、融资和基建能力水平。建立市县园区人才与项目互联互通平台。

　　培育壮大主导产业。做大增量、优化存量，推动七大主导产业集聚发展。围绕产业、资源、劳动力和乡贤等优势，加大产业招商力度。注重项目谋划，抓好重点专项规划对接，争取更多项目进入上级规划“笼子”。开工建设粤西LNG造陆工程。大力支持茂名石化新上项目、粤桥矿业30万吨/年钛毛矿精加工、六韬珠宝等项目建设。强化“一事一策一团队”工作机制。深入推进领导干部挂钩服务企业活动。全面落实企业减负政策和工业技改扶持政策。完善技改（创新）重点项目储备库。研究出台技改事后奖补实施细则。积极组织企业申报省级技改专项资金。加大规模企业奖励、品牌奖励、投融资服务、用地（林、海）扶持等力度。加快实施重点骨干企业培育计划，各区（市）重点培育2-3家行业龙头企业。

　　加快发展电子商务。全面落实《电商发展三年行动计划（2016-2018年）》。培育扶持一批青年创业者、中小微企业、专业市场、批发零售行业等电商经营主体。通过牵手电商巨头、培育本土化平台、企业自建平台等方式，加快完善网络销售渠道。挖掘特色产品优势，注重原产地品牌建设和产品质量管理，扩大特色产品网上销售。各区（市）加快建成至少一个特色鲜明的电商集聚区或物流园。推进信息基础设施建设、快递下乡工程和电商服务业，进一步完善电商服务体系。

抓好房地产业、旅游业和新兴产业发展。落实国家出台的房地产去库存系列政策措施。适度开发旅游地产、养老地产、商业地产。积极开展包茂高速沿线城市旅游合作及营销。精心组织好“开渔节”。支持浪漫海岸国际旅游度假区三期、御水古温泉度假村二期、放鸡岛海洋度假公园扩建改造项目建设。支持南海旅游岛按照国家4A景区标准进行规划和改造。加快推进旅游厕所建设改造及管理。大力发展新兴产业，推进光伏发电、绿色能源发电和充电桩等项目建设。

（三）突出抓好中心城区扩容提质，加快建设粤西组团式中心城市。

　　加强北优组团建设。加快完善东北五小区、油十片区、站南片区及西城片区路网和市政设施。建成文明北路（官渡路至大园路段）、大园三路。统筹推进站南片区西粤南路、站前大道、站南路、金城路及排水排污工程建设。完成站前路、江东路等沥青罩面工程。加快青少年活动中心建设。着力推进露天矿“引水、种树、建馆、修路”四大工程。加快建设官渡公园、上宾体育健身公园及若干街头公园。推进小东江综合整治，建设生态休闲公园。升级改造城区公园、广场、游乐场。加快农贸市场改造和布局。大力实施中心城区亮化工程。抓好棚户区改造和河西老工业区搬迁改造。做好迎接国家园林城市复检工作。

　　推动中联组团发展。推进国家新型城镇化试点工作。抓紧完成市民大道二期、高新大道二期建设。积极推进市民大道三期建设。加快粤西农批、亿丰家居、华南商贸城、市交投物流园等商贸物流重点项目建设。利用好市民中心和市民公园，吸引发展要素集聚。推进颐年苑等项目建设。

加快南拓组团建设。加快推进电白城区“七路一桥一广场”建设，动工建设兴华路、登步路、水东大道镇东段，扩建迎宾北路。建成包茂大道南。加强城区防涝排污系统改造。加快水东湾综合整治开发项目，力争年内完成大桥主桥桩。

加快南海半岛绿道和道路两侧改造建设。力争完成海洋公园（一期）工程。加快推进高地智慧城项目，抓好路网工程、广科院滨海校区、市中心医院、歌美海公园等项目建设。

　　推进东进组团建设。完成茂名港大道改线和延长线、博贺湾大道、G325电城段路面改造、Y880恒大路至管委会段、污水处理厂（首期）工程。基本完成博贺湾东片区（一期）土地一级开发。加快保利大都会、新渔港、安置区建设。开工建设博贺湾大桥及东连接线、东湾公租房、高州人民医院滨海分院。加快建设滨海新区供水工程。

　　提高城市精细化管理水平。继续推进城市管理综合行政执法体制改革。实施数字城管建设工程。深入落实“门前三包”责任制。推动城区环卫实现社会化、市场化管理。启动国家卫生城市创建工作。落实家禽“集中屠宰、冷链配送、生鲜上市”工作。加强对小街小巷硬底化改造及交通标识设置，分散主干路交通压力。加强户外广告、公共停车位和小区物业经营管理。

　　（四）突出抓好特色县域经济，统筹城乡协调发展。

　　积极推进农业现代化。加快高标准基本农田建设。扶持发展一批特色农产品基地、农业专业村镇建设。加强特色果蔬等大宗农产品主产区建设。完善农业科技推广服务体系。实施农民合作社提升工程。抓好渔船更新改造工作。积极开展农产品促销，充分发挥粤西农批作用。强化名特优新农产品品牌培育与建设。扩大政策性农业保险覆盖面。

　　全力推进县域产业发展。各区（市）结合产业功能定位和发展实际，着力培育主导产业。扶持信宜电子元件生产及矿产加工、高州不锈钢制品及皮革生产、化州生物医药及矿产加工、茂南石化及粮食饲料生产加工、电白水海产品加工及香精香料等产业发展。谋划建设一批亿元以上特色产业项目。

　　构建布局合理的城镇体系。抓好高州、信宜和化州等次中心城市和中心镇建设。信宜重点推进城东站前片区、城西片区、行政中心区、城南一河两岸商住区、达北线商贸新区建设，高州按照“优化老城，东拓新区，北跨鉴江，南联金山”思路推动形成“一城多区”格局，化州积极拓展橘州新城居住商贸片区、城南金融商贸片区、城西标志性商住小区、城东行政文体片区。推进“三旧”改造、城镇棚户区和城乡危房改造。

　　全面推进新农村建设。积极探索农村综合改革。编制完善县级市全域城乡建设规划，提高村庄规划覆盖率，强化规划执行率。全面推进农村土地承包经营权确权登记颁证工作。健全覆盖县镇村的农村产权流转管理服务平台体系。深入实施城乡清洁工程。建设茂名特色美丽乡村。推进名镇名村、幸福村居、文明示范村建设，加强历史文化名镇名村名居和传统村落保护。推进信宜、茂南和化州3个省级新农村示范片建设。认真开展第三次全国农业普查工作。

　　（五）突出抓好重点领域改革，推动形成发展新动力。

　　深入推进行政体制改革。健全市县政府部门权责清单制度，制定镇街权责清单。深化行政审批制度改革。推进国有林场、农垦、供销社、市直高等中等职业院校等事业单位重点领域改革。统筹推进社会信用信息平台和市场监管平台建设。加快推动基层公共服务综合平台和“一门式网格化”全科政务服务创新体系建设。推进市公共服务中心实体大厅、网上办事大厅以及行政审批与公共资源交易代理“中介超市”建设。

　　积极推进经济领域改革创新。深化财政体制改革，完善扩大零基预算试点，研究编制三年滚动预算和镇级基层运转补助经费使用办法。压减专项转移支付，盘活财政存量资金统筹用于重点领域项目建设。加强重大项目资金管理和政府性债务管理。深入推进农村普惠金融试点工作。力争年内完成1家农信联社向农村商业银行转制改革。推进中小企业“新三板”、“四板”挂牌融资。深化国资监督管理体制改革。谋划组建市金融控股集团公司及政策性融资担保公司。深化国企负责人薪酬制度改革。健全国有公司法人治理结构。积极推广PPP融资新模式。加快推进公共资源交易改革。

　　促进民营经济加快发展。坚持市场化改革导向，为民营经济创造宽松的发展环境和足够的发展空间，鼓励民营企业进入更多竞争性领域。加强对民营企业家的正面宣传，鼓励民营企业家创业创新、依法经营和回报社会，在全社会营造崇尚创业、支持创业、勇于创业的良好社会氛围。

　　加强对外合作交流。抢抓“一带一路”、包茂高速开通机遇，推进茂名梧州战略合作和泛北部湾城市群合作，加快茂名向东向北区域合作发展。切实做好对台工作。积极邀请外地茂名商会回茂、海外侨商组团来茂考察访问。加强与东南亚经济文化交流。

（六）突出抓好环保生态建设，推动绿色发展。

　　加强环境综合治理。加快工业生活污水处理设施及配套管网建设与改造。建成茂南区污水处理厂（一期）和水东湾新城水质净化厂项目。加快14个中心镇污水厂建设，规划建制镇污水处理设施建设。强化饮用水源地水质保护长效机制。落实好“河长制”水环境监测工作。加快推进高州水库除险加固工程恢复89米正常蓄水位征地移民搬迁安置项目。加强小东江、鉴江、袂花江等重点河流污染治理，提高交界断面水质达标率。加快禁养区规模化畜禽养殖场核准清理和非禁养区养殖场限期整治工作。抓好重点区域和特殊天气空气质量监控。积极争取上级支持，推进炼油厂卫生防护距离内居民搬迁安置工作。

　　推进资源节约利用。加快淘汰“黄标车”和燃煤锅炉。严格落实节能目标责任制。实施重点节能工程。加大淘汰落后产能工作力度。加快推进循环经济试点示范体系建设。

筑牢生态安全屏障。加强沿海防风林、红树林及河口湿地生态功能区保护。开展海岸带和海岛综合治理修复，推进美丽海湾建设。推进森林进城围城工程。建成20个乡村绿化美化示范点，实现村庄绿化覆盖率达30%以上。抓好G207和G325等重要道路两侧生态景观林带建设。加快推进高州水库集水区生态公益林全覆盖。严厉打击违法侵占林地行为。

（七）突出抓好民生事业，推动共享发展。

　　推进就业社保扶贫工作。认真落实“双创”战略，深入实施“千人创业带动万人就业工程”，全年新增城镇就业6万人，促进创业2200人，新增转移就业劳动力5.5万人，转移劳动力技能培训8000人。抓紧出台急需紧缺人才管理办法。巩固城镇职工基本养老保险扩面征缴成果。完善城乡居民大病医疗保险制度和城镇职工补充医疗保险制度。加快征地农民社保资金分配工作。加大保障房建设力度。实施精准扶贫，启动第三轮扶贫开发“双到”工作。

　　统筹推进科教文卫事业发展。深入实施创新驱动发展战略，加快培育科技企业孵化器。完成教育强市督导验收和国家义务教育发展基本均衡县督导评估工作。启动创建教育现代化先进市工作。建成愉园小学和市中小学信息技术教育中心楼。抓好市育才小学、崇文学堂等学校建设工作。加强中职综合实训中心和县域职教中心建设。推动现代职业教育综合改革示范市创建工作。高度重视“一本五专”建设，启动广东石油化工学院西城校区建设，抓好广东茂名幼儿师范专科学校、广东农林科技职业学院筹建工作。大力支持特殊教育和民办教育。启动创建卫生强市工作。深化县级公立医院改革，做好城市公立医院改革试点。规范、促进民营医疗机构发展。加快推进中医药强市建设。抓好一对夫妇可生育两个孩子政策实施。推进现代公共文化服务体系建设和全民健身活动。深入推进文化惠民工程。

加强和创新社会治理。深入推进平安茂名建设。严防妥处暴恐活动，重拳打击电信诈骗、涉枪、涉毒等各类突出犯罪。建立和完善立体化社会治安防控体系。加强互联网安全管理。强化食品药品安全重点整治。严格落实安全生产责任，坚决遏制重特大安全事故。提升突发事件应急处置能力。

（八）突出抓好政府自身建设，确保工作落实。

　　加强民主法治建设。认真落实市人大及其常委会决议和决定。主动加强与政协沟通联系。认真办理人大代表建议和政协委员提案。广泛听取各民主党派、工商联、无党派人士和人民团体意见。主动向社会公开重要民生事项。健全依法行政决策机制，落实《茂名市重大决策社会稳定风险评估工作规程》。建立健全政府立法制度和程序，提高依法行政水平。加大群众网上诉求办理力度。抓好镇村两级人民调解委员会规范化建设、“七五”普法、“一村（居）一法律顾问”和信访维稳工作。

　　大力弘扬实干精神。坚持重心前移、主动服务、层层传压，形成你追我赶、争先进位的良好氛围。强化责任担当，任务细化到点，责任落实到人，敢于啃“硬骨头”，用实绩来检验落实效果。坚持问题导向，打破陈规陋习，鼓励创新，宽容失败。发扬“钉钉子”精神，牢牢盯住重点工作和重点项目，实实在在把一件件事情干好。继续开好县域发展交流会和三大平台建设工作现场会。强化考核激励和督查问责。

　　更加注重廉政建设。深入践行“三严三实”要求，不断加强作风建设。严格落实《中国共产党廉洁自律准则》。认真落实中央八项规定，防止“四风”问题反弹。强化经济责任审计。严格落实“一岗双责”。健全廉政风险防控体系，强化源头治理和制度防腐。加强行政问责和执法监察。

　　各位代表，茂名加快发展的态势已经形成，未来五年的发展蓝图已经绘就。站在新起点上，让我们紧密团结在以习近平同志为总书记的党中央周围，在省委、省政府和市委的正确领导下，坚定信心，抢抓机遇，咬定目标，扎实苦干，不断谱写茂名振兴发展新篇章！
